# Supplementary material for: Modelling Skylarks (Alauda arvensis) to Predict Impacts of Changes in Land Management and Policy: Development and Testing of an Agent-Based Model
Source: PLoS One. 2013 Jun 6;8(6):e65803. doi: 10.1371/journal.pone.0065803 (PMC3675089; doi:10.1371/journal.pone.0065803)
Supplement: Supporting Information S4 — The skylark ODdox as a zipped archive. (ZIP) [file pone.0065803.s004.zip › Skylark_ODdox/structa_date.html]

ALMaSS Skylark ODdox: aDate Struct Reference


ALMaSS Skylark ODdox
 2.0


- Main Page
- Related Pages
- Classes
- Files

- Class List
- Class Index
- Class Hierarchy
- Class Members

Public Attributes

aDate Struct Reference

`#include <calendar.h>`

List of all members.

|  |  |
| --- | --- |
| Public Attributes | |
| int | day |
| int | month |
| int | year |

---

## Member Data Documentation

int aDate::day

int aDate::month

int aDate::year

---

The documentation for this struct was generated from the following file:

- calendar.h


- aDate
- Generated on Thu Jan 10 2013 13:15:35 for ALMaSS Skylark ODdox by
   1.8.1.1
